# Poly-Epsilon-Lysine Hydrogels with Dynamic Crosslinking Facilitates Cell Proliferation

**DOI:** 10.3390/ma13173851

**Published:** 2020-09-01

**Authors:** Nestor Lopez Mora, Matthew Owens, Sara Schmidt, Andreia F. Silva, Mark Bradley

**Affiliations:** 1 EaStCHEM School of Chemistry, The University of Edinburgh, Edinburgh EH9 3FJ, UK; matthew.simmonte@ed.ac.uk (M.O.); ask.schmidt@gmail.com (S.S.); 2 School of Physics and Astronomy, The University of Edinburgh, Edinburgh EH9 3FD, UK; andreia.silva@ed.ac.uk

**Keywords:** dynamic hydrogels, poly-ε-lysine, RGD peptide, imine crosslinking, 4-arm PEG

## Abstract

The extracellular matrix (ECM) is a three-dimensional network within which fundamental cell processes such as cell attachment, proliferation, and differentiation occur driven by its inherent biological and structural cues. Hydrogels have been used as biomaterials as they possess many of the ECM characteristics that control cellular processes. However, the permanent crosslinking often found in hydrogels fails to recapitulate the dynamic nature of the natural ECM. This not only hinders natural cellular migration but must also limit cellular expansion and growth. Moreover, there is an increased interest in the use of new biopolymers to create biomimetic materials that can be used for biomedical applications. Here we report on the natural polymer poly-ε-lysine in forming dynamic hydrogels via reversible imine bond formation, with cell attachment promoted by arginine-glycine-aspartic acid (RGD) incorporation. Together, the mechanical properties and cell behavior of the dynamic hydrogels with low poly-ε-lysine quantities indicated good cell viability and high metabolic activity.

## 1. Introduction

Hydrogels are highly hydrated three-dimensional (3D) polymer networks that have been used for a broad range of biomedical applications that range from tissue engineering [1,2] and surgical glues [3,4] to contact lenses [5,6], materials for drug delivery [7], and biosensors [8]. In large part this is due to their tunable mechanical properties and their biocompatibility [9]. The high water content of these 3D macromolecular networks and the ability to decorate them with ligands creates an ideal environment for diffusion and transport of nutrients, while offering optimal characteristics for generating a 3D cell culture matrix. However, the high degree of crosslinking in hydrogels, necessary to provide stability and structural support to cells, creates a static polymer network that hinders cell migration, a critical feature of the dynamic environment of the natural extracellular matrix (ECM) [10]. Cell migration has been achieved by the introduction of hydrogel degradability with, for example, the incorporation of hydrolytically degradable polylactide segments [11,12], or substrates of degradative enzymes such as metalloproteases [13,14], but in these cases the material is permanently broken down leading to material collapse over time. Recently, using dynamic covalent chemistry [15], hydrogel networks with exchangeable, reversible, or adaptable linkages have been formed through Diels-Alder [16], hydrazine [17], Schiff’s base (imine) [18], oxime [19], and disulfide [20] bond formation. The dynamic bond breakage and reformation generates a rearranging molecular network that allows cells to move and spread throughout the 3D polymer network [21], opening a new set of potential properties such as self-healing [18,22], shape memory [23], and stimuli-responsiveness [24,25,26], enhancing their potential use in biomedical applications.

ECM mimics have been successfully generated using numerous synthetic polymers, creating hydrogels with unique structural and mechanical properties for use as cellular supports [27]. In the majority of cases the desired characteristics include controllable matrix stiffnesses and cell biocompatibility, resulting in optimal cell adhesion and survival [28]. Despite the advances in synthetic biomaterials, there is considerable interest in using naturally occurring polymers to create biomimetic materials for biomedical applications. Biopolymers previously explored include chitosan [29,30], alginate [31,32], gelatine [33], and hyaluronic acid [34], which have been functionalized and used as biopolymer precursors to form dynamic hydrogels. This creates a responsive structure akin to the natural ECM and allows for in situ cell encapsulation. The hydrogel/cell association can be further enhanced with the incorporation of peptides [35], proteins such as growth factors [36], polysaccharides and proteoglycans [37], or synthetic analogues, thus allowing biological properties such as cell attachment, proliferation, and differentiation to be introduced and modulated. The peptide motif arginine-glycine-aspartic acid (RGD) is a peptide adhesion sequence found in many ECM proteins such as fibronectin, fibrinogen, vitronectin, and laminin as a specific integrin ligand [38,39,40]. Many aspects of the RGD motif such as structure [41,42], spacing [43], and density distribution [44,45,46] have been studied, with synergism between the biomechanical properties of the polymer matrix and the RGD motif modulating the cellular adhesive response. Additionally, plasma protein binding onto RGD-functionalized biomaterials further passivates the surface and promotes cellular binding.

Poly-ε-lysine is a natural homo-poly-amino acid used as an emulsifier and preservative in the food industry (with FDA certification [47]) that is nontoxic towards humans [48,49], and has been demonstrated to be biocompatible [50]. Herein, the naturally occurring poly-ε-lysine, without further functionalization, was exploited in the design of arrays of dynamic hydrogels that were crosslinked via reversible Schiff-base bond formation with a 4-armed PEG-aldehydes. There are a limited number of studies with hydrogels including poly-ε-lysine [51], or modified versions of poly-ε-lysine [52], to form conventional chemically crosslinked hydrogels, for example via amide bond crosslinking. To the best of our knowledge, however, no designed dynamic poly-ε-lysine hydrogels have been proposed as ECM mimics [53]. This is perhaps due to the relative weakness of the reversible crosslinking that produces softer hydrogels in comparison to stiff static hydrogels produced with conventional chemical crosslinking. Contrary to our initial hypothesis that the high amount of amine groups distributed along the poly-ε-lysine backbone would be detrimental to cell viability, high cell viability was observed on the poly-ε-lysine dynamic hydrogels (HG-PεK) formulated at low ratios of poly-ε-lysine. Additionally, the linear poly-ε-lysine backbone led to a suitable network with the required biomaterial stiffness to afford cell structural support and proliferation. To aid cellular adhesion and binding specificity, the linear peptide H-Ahx-GRGDSK-NH_2_ (referred to here as RGD) with primary amines at both the amino and carboxyl termini (via the lysine residue) was incorporated during hydrogel formulation. An RGD linear structure was selected over the cyclic analogue based on a previous report that showed improved long-term cellular adhesion [54]. A range of RGD concentrations was explored to determine cellular adhesion and survival on HG-PεK. The HG-PεK was compared to a similar dynamic hydrogel made of the non-fouling polymer poly(ethylene glycol) (HG-PEG) to isolate the RGD contribution to cell binding properties, with higher performance found for HG-PεK compared to HG-PEG.

## 2. Materials and Methods

Materials. The 4-arm PEG-OH (10,000 Da) and H_2_N-PEG-NH_2_ (2000 Da) were purchased from JenKem Technologies (Plano, TX, USA). Poly-ε-lysine (3500–4000 Da) was purchased from Carbosynth (Compton, UK). All amino acids, aminomethyl polystyrene resin, and the Fmoc-Rink amide linker were purchased from GL Biochem (Shanghai) Ltd. (Shanghai, China) or NovaBiochem (Merck, Darmstadt, Germany). All other chemicals were purchased from Sigma Aldrich (St. Louis, MO, USA) or Acros Organics N.V. (Geel, Belgium) and used without further purification, unless otherwise indicated. Dulbecco’s Modified Eagle Medium (DMEM), LIVE/DEAD Cell Imaging Kit (488/570 nm), and AlamarBlue^®^ cell viability reagent were purchased from Thermo Fisher (Waltham, MA, USA). Phosphate-buffered saline (PBS) was purchased from Sigma Aldrich (St. Louis, MO, USA) DMEM was prepared with 10% fetal bovine serum (FBS), 100 U/mL penicillin, 100 µg/mL streptomycin, and 2 mM glutamine (referred to as complete DMEM). The 4-arm PEG-aldehydes with >90% end group functionalization were synthesized following a method reported elsewhere (see Figures S1 and S2 in the Supplementary Materials) [55]. HeLa cells were purchased from American Type Culture Collection (Manassas, VA, USA) and cultured in 25 cm^2^ flasks (Corning) using complete DMEM, with passage every 2–3 days using Trypsin-EDTA (Sigma).

RGD solid-phase synthesis. Linear H-Ahx-GRGDSK-NH_2_ with a 6-aminohexanoic acid (Ahx) spacer at the N-terminus was synthesized on amino methyl polystyrene resin and functionalized with an Fmoc-Rink linker, using Fmoc/*t*Bu solid-phase synthesis. The peptide was cleaved for 3 h in a cleavage cocktail of 95% trifluoroacetic acid (TFA), 2.5% triisopropylsilane, and 2.5% water with constant mixing at room temperature. The peptide was precipitated from the filtrate using cold diethyl ether, collected by centrifugation, and dried under vacuum. The RGD peptide was dissolved in 0.1% formic acid in water and purified by reversed-phase flash chromatography (Biotage Isolera, Uppsala, Sweden) with a SNAP Ultra C18 column (Biotage, Uppsala, Sweden). The solvents consisted of a mixture of 95% water/5% acetonitrile (0.1% formic acid: solvent A) and 100% acetonitrile (solvent B). A gradient of solvent B from 0 to 20% in 20 min, 20 to 95% in 5 min, and 95% for 1 min was used for peptide purification. The appropriate fractions were detected at λ = 214 nm, combined, concentrated, and lyophilized. The peptide was characterized by analytical C18 reversed-phase HPLC (Agilent 1100 ChemStation, Santa Clara, CA, USA) with an RGD peptide purity of >95% and HRMS (RGD m/z calculated 730.4086, m/z found 730.4127).

Hydrogel formation. Hydrogels were prepared by dynamic covalent crosslinking via Schiff’s base formation. Stock polymer solutions were prepared by dissolving the 4-arm PEG-aldehydes, poly-ε-lysine or H_2_N-PEG-NH_2_ to complete dissolution in PBS (pH 7.4) at room temperature. The hydrogel poly-ε-lysine (HG-PεK) was prepared in 500 µL batches varying the molar ratios of the 4-arm PEG-aldehydes and the lysine unit (in the poly-ε-lysine) (1:2 and 1:10) with a final polymer precursor weight of 10% *w*/*v*.

Thus, the HG-PεK with a molar ratio of 1:2 was prepared by mixing stock solutions of 4-arm PEG-aldehydes (250 µL, 20% *w*/*v*) with poly-ε-lysine (100 µL, 5% *w*/*v*) and 150 µL PBS. The HG-PεK (molar ratio 1:10) was prepared by mixing stock solutions of the 4-arm PEG-aldehydes (250 µL, 20% *w*/*v*) with poly-ε-lysine (130 µL, 20% *w*/*v*) and 120 µL PBS.

In a similar manner, a non-fouling hydrogel was prepared by mixing 4-arm PEG-aldehydes (250 µL 20% *w*/*v*) with H_2_N-PEG-NH_2_ (200 µL, 20% *w*/*v*) and 50 µL PBS. Following the same procedures, hydrogel constructs incorporating RGD were prepared at final concentrations of 0, 0.2, 2, 4, 6, and 20 mM RGD with the addition of the stock RGD solution (100 mM in PBS) (see Tables S1–S3 in the Supplementary Materials). The pH of the final hydrogel solutions was measured immediately after mixing the precursor stock solutions with pH paper and before gelation. Gelation occurred within 2 to 6 h at room temperature.

Rheological characterization. Small amplitude oscillatory shear (SAOS) measurements were performed in duplicate using a strain-controlled ARES-G2 Rheometer (TA Instruments, New Castle, DE, USA). A sandblasted plate-plate geometry (40 mm, stainless steel) with a gap of 300 µm was used. Hydrogel samples of 500 µL were measured. Time sweeps were performed at an angular frequency of 1 Hz and constant strain of 1% at 37 °C. Amplitude sweep experiments were performed for strains (γ) between 0.01 and 100% at constant frequency (ω = 10 rad/s), and it was found that for a strain of 1% both moduli were in the linear viscoelastic region (LVE). Frequency sweeps were performed with a constant strain of 1% for angular frequencies between 0.1 and 100 rad/s at 37 °C.

Cryo-Scanning Electron Microscopy (cryo-SEM). The internal structure of the hydrogel constructs was imaged in a Gemini 2 FIB-cryo-SEM (Zeiss, Jena, Germany). The hydrogel construct was placed in the sample holder and frozen with liquid nitrogen. The hydrogel was then freeze fractured with a scraper, sputtered with platinum, and imaged at 3.0 kV.

Cell culture conditions. A 96-well plate was passivated with a stock solution of poly-L-lysine (0.01% *w/v*), incubated for 10 min, drained, and dried overnight at room temperature. Hydrogel arrays of HG-PεK and HG-PEG with RGD concentrations of 0, 0.2, 2, 4, 6, and 20 mM were made by transferring 50 µL of each hydrogel construct into the previously functionalized 96-well plate or 10 µL on an Ibidi µ-Slide Angiogenesis well plate (without surface passivation). After hydrogel formation, the 96-well plate or Ibidi µ-Slide Angiogenesis well plate holding the hydrogel array was sterilized by UV light for 60 min, before rinsing the chambers with complete DMEM. HeLa cells were seeded at a density of 2 × 10^4^ cells per well in a 96-well plate or a density of 2.7 × 10^3^ HeLa cells per well in the Ibidi µ-Slide Angiogenesis well plate, and incubated at 37 °C, 5% CO_2_. Cell culture was maintained with complete DMEM exchanges every 48 h.

Live/Dead cell viability analysis was performed using calcein AM for a live stain (λ_ex_/λ_em_ = 488/515 nm) and propidium iodide as a dead stain (λ_ex_/λ_em_ = 570/602 nm) after 48 h of in vitro cell culture in HG-PεK or HG-PEG with and without RGD. Fluorescence imaging was performed on a Zeiss AxioVert 200M fluorescence microscope for the 96-well plate hydrogel and laser scanning confocal microscopy on a Leica TCS SP8 confocal (CALM facilities, Queen’s Medical Research Institute, Edinburgh, UK) for the Ibidi µ-Slide Angiogenesis well plate and analyzed using the software Fiji-ImageJ [56].

AlamarBlue proliferation assays were performed in 96-well plates holding the hydrogel construct array with measurement of AlamarBlue fluorescence after HeLa cell culture. HeLa cells were seeded in HG-PεK and HG-PEG with and without RGD at a cell density of 2.0 × 10^4^ cells per well and incubated with complete DMEM for 48 h at 37 °C and 5% CO_2_. Subsequently, DMEM was removed and AlamarBlue (10% *v*/*v*, 200 µL) in medium was added. An immediate baseline reading of fluorescence was taken at 590 nm using a BioTek HT Synergy multimode reader (Winooski, VT, USA) with Gen 5.2.06.10 software (BioTek Instruments, Winooski, VT, USA). The cell seeded gels were then incubated for 5 h and remeasured for fluorescence, with subtraction of the baseline measurement per well. Cell numbers were quantified using a calibration curve displaying fluorescence as a function of cell number prepared with HeLa cells seeded in 96-well tissue culture plastic plates at densities of 6.0 × 10^4^, 3.0 × 10^4^, 1.2 × 10^4^, 6.0 × 10^3^, 3.0 × 10^3^, and 0.6 × 10^3^ cells per well incubated at 37 °C, 5% CO_2_ (Figures S3–S5 in the Supplementary Materials).

Statistical analysis was performed by one-way ANOVA followed by Bonferroni’s post-test using Graphpad Prism 5.0 (San Diego, CA, USA). A *p* < 0.05 was considered statistically significant. Results are presented as mean ± standard deviation. Three independent biological experiments were prepared for each experiment.

## 3. Results and Discussion

The well-defined lengths, ready functionalization of the terminal sites, and varied architectures made the multi-arm PEG architectures attractive scaffolds for the design and fabrication of a wide variety of hydrogels with different crosslinking chemistries [57]. Herein, the hydrogel precursor 4-armed PEG-aldehydes were prepared by nucleophilic substitution of commercially available 4-arm PEG-OH with 2-bromo-1,1-diethoxyethane, followed by acid hydrolysis of the acetal to yield the corresponding aliphatic 4-arm PEG-aldehydes.

**Figure 1:**
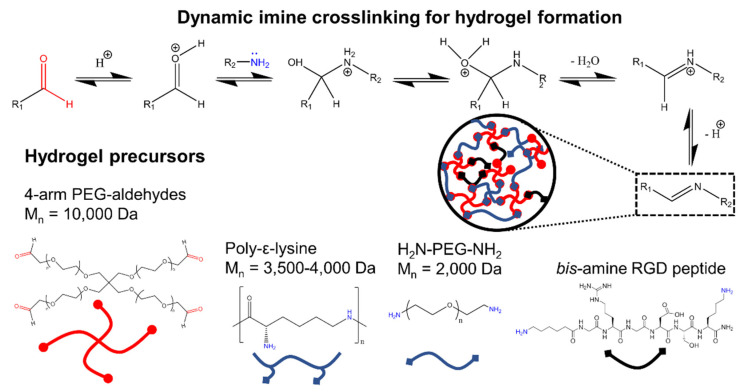
Hydrogels generated via dynamic imine crosslinking. The hydrogels were fabricated using a 4-arm PEG each terminating in an aldehyde group and mixed with either poly-ε-lysine to give HG-PεK or a linear diamine PEG to give HG-PEG. The peptide arginine-glycine-aspartic acid (RGD) was synthesized as a *bis*-amine and was added at differing concentrations to promote cell binding.

**Figure 2:**
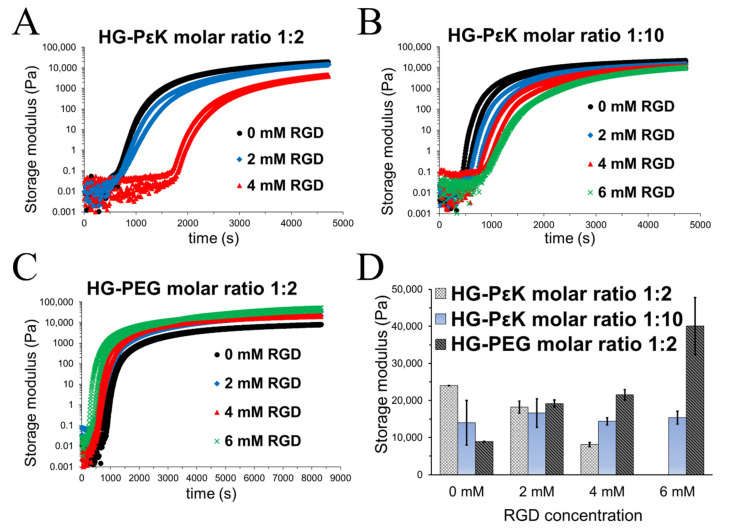
Hydrogel mechanical characterization by oscillatory rheology. Time sweep plots are displayed as two independent flow traces for each hydrogel construct as a function of RGD concentration at 37 °C. (**A**) Time sweep for HG-PεK molar ratio 1:2 with 0 mM RGD (black circle), 2 mM RGD (blue diamond), and 4 mM RGD (red triangle). (**B**) Time sweep for HG-PεK molar ratio 1:10 with 0 mM RGD (black circle), 2 mM RGD (blue diamond), 4 mM RGD (red triangle), and 6 mM RGD (green cross). (**C**) Time sweep for HG-PEG molar ratio 1:2 with 0 mM RGD (black circle), 2 mM RGD (blue diamond), 4 mM RGD (red triangle), and 6 mM RGD (green cross). (**D**) Storage moduli as a function of RGD concentration at 37 °C. The storage moduli were obtained at 1 Hz from frequency sweeps in the linear viscoelastic region presented in Figures S8–S10 in the Supplementary Materials. Data represent mean ± standard deviation (n = 2).

The 4-arm PEG-aldehydes precursor was mixed with poly-ε-lysine at molar ratios 1:2 and 1:10, resulting in reversible crosslinking via imine formation in PBS (see Figure 1). Hydrogels were formed with concentrations of 4-armed PEG-aldehydes above 5% *w*/*v* at both molar ratios (see Figure S6 in Supplementary Materials). Similarly, 4-armed PEG-aldehydes were mixed with diamino-PEG to produce hydrogels at precursor concentrations above 8% *w*/*v* (see Figure S7 in Supplementary Materials). Generally, a faster gel formation was observed with higher 4-armed PEG-aldehydes precursor concentrations. The cell adhesive peptide motif RGD was synthesized in such a way that it would be incorporated into the hydrogel network as an additional crosslinker via dynamic imine bond formation with concentration levels between 0 and 20 mM. Gel formation was observed for all RGD constructs, but slower gelation was observed for gels with higher levels of RGD (see Figure 2A–C). For example, HG-PεK with a molar ratio of 1:2 with 0.2 mM RGD produced a gel after 2 h, while with 20 mM gelation took over 6 h at room temperature.

**Figure 3:**
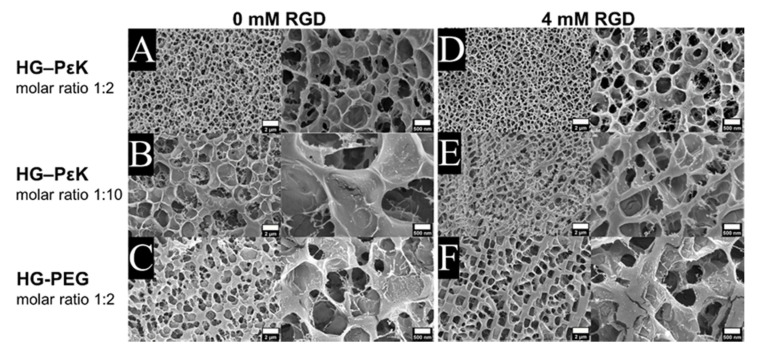
Hydrogel internal structures imaged by cryo-SEM. (**Left**) Hydrogel morphology without the RGD peptide for (**A**) HG-PεK molar ratio 1:2, (**B**) HG-PεK molar ratio 1:10, and (**C**) HG-PEG molar ratio 1:2. (**Right**) Hydrogel morphology with 4 mM RGD peptide for (**D**) HG-PεK molar ratio 1:2, (**E**) HG-PεK molar ratio 1:10, and (**F**) HG-PEG molar ratio 1:2. For each panel the scale bars in the left micrographs are 2 µm and right micrographs are 500 nm.

The internal structure of the hydrogel was imaged by cryo-SEM, with the microstructure of HG-PεK and HG-PEG examined in the swollen state and compared to that of hydrogels containing the cell adhesive peptide RGD at 4 mM. Overall, all the cryo-dried hydrogels displayed a honeycomb-like 3D network structure with some notable differences in pore size. Smaller pores were observed for HG-PεK in comparison to HG-PEG for constructs with a molar ratio 1:2 (Figure 3A,C). The addition of RGD had no visible effect on the hydrogel microstructure of either HG-PεK or HG-PEG (Figure 3D,F). Increasing the PεK molar ratio to 1:10 in HG-PεK (Figure 3B) resulted in larger pore sizes than that of HG-PεK with a molar ratio of 1:2. Here, the addition of RGD had a clear effect on the microstructure of HG-PεK molar ratio 1:10, yielding a more compact microstructure with smaller pore sizes (Figure 3E).

Oscillatory rheology time and frequency sweeps were performed to provide insight into the gelation time and stiffness of HG-PεK and HG-PEG as a function of molar ratio and RGD concentrations. HG-PεK with a molar ratio of 1:2 in the absence of RGD had a delay in gelation rate compared to HG-PεK with a molar ratio of 1:10. This increase in gelation time presumably arises due to fewer free poly-ε-lysine chains available during the reversible crosslinking. The sequential increase of RGD concentration in HG-PεK at both molar ratios led to weaker network structures with slower gelation rates. This behavior was more evident at molar ratios of 1:2, probably due to the competition of amine groups from the RGD and the poly-ε-lysine chains for reversible imine bond formation (Figure 2A,B). On the other hand, the opposite behavior was observed for the HG-PEG gels with rapid gelation time upon RGD addition (Figure 2C).

The storage moduli was obtained from frequency sweeps with a fixed strain of 1% (within the linear viscoelastic region) as a function of RGD concentration (Figures S8–S10 in Supplementary Materials). RGD negligibly contributed to the storage moduli in HG-PεK with a molar ratio of 1:10 due to excess of poly-ε-lysine in the hydrogel formulation. In contrast, HG-PεK and HG-PEG with a molar ratio of 1:2 showed distinct and opposing storage moduli trends as a function of RGD concentration. While the storage moduli decreased in HG-PεK, the storage moduli in HG-PEG increased with increases in RGD levels (Figure 2D). Overall the mechanical properties and cryo-SEM analysis agreed with the higher storage moduli and the compact microstructure in HG-PεK with a molar ratio of 1:2, compared to the lower storage moduli and larger pore sizes found in HG-PεK with a molar ratio of 1:10. On the other hand, the increase in the concentration of RGD led to significant differences in HG-PEG storage moduli in both the absence and presence of RGD, despite similar internal structure and pore size. Regardless of the opposite mechanical properties between HG-PεK and HG-PEG, the constructs had the same storage moduli when formulated at 2 mM RGD, indicative of similar material stiffnesses at this peptide concentration.

**Figure 4:**
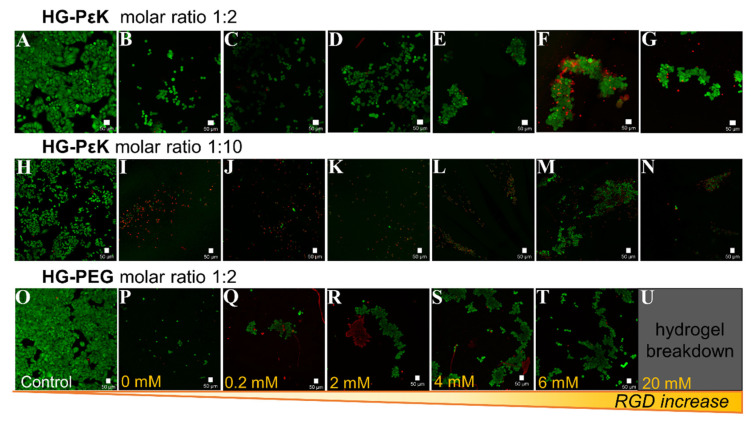
Live/dead cell viability staining of HeLa cells seeded on a dynamic hydrogel array with varying levels of the peptide RGD. HeLa cells were seeded and incubated with complete DMEM for 48 h at 37 °C and 5% CO_2_ before imaging on (**A**,**H**,**O**) tissue culture plate, (**B**) HG-PεK molar ratio 1:2 with 0 mM RGD, (**C**) HG-PεK molar ratio 1:2 with 0.2 mM RGD, (**D**) HG-PεK molar ratio 1:2 with 2 mM RGD, (**E**) HG-PεK molar ratio 1:2 with 4 mM RGD, (**F**) HG-PεK molar ratio 1:2 with 6 mM RGD, (**G**) HG-PεK molar ratio 1:2 with 20 mM RGD, (**I**) HG-PεK molar ratio 1:10 with 0 mM RGD, (**J**) HG-PεK molar ratio 1:10 with 0.2 mM RGD, (**K**) HG-PεK molar ratio 1:10 with 2 mM RGD, (**L**) HG-PεK molar ratio 1:10 with 4 mM RGD, (**M**) HG-PεK molar ratio 1:10 with 6 mM RGD, (**N**) HG-PεK molar ratio 1:10 with 20 mM RGD, (**P**) HG-PEG molar ratio 1:2 with 0 mM RGD, (**Q**) HG-PEG molar ratio 1:2 with 0.2 mM RGD, (**R**) HG-PEG molar ratio 1:2 with 2 mM RGD, (**S**) HG-PEG molar ratio 1:2 with 4 mM RGD, (**T**) HG-PEG molar ratio 1:2 with 6 mM RGD, and (**U**) HG-PEG molar ratio 1:2 with 20 mM RGD. Live cells are in green and dead cells are in red. Scale bars are 50 µm.

HG-PεK and HG-PEG constructs were interrogated for their ability to allow cellular adhesion and proliferation as a function of RGD concentration using confocal microscopy (Figure 4). HeLa cells, a common and widely laboratory available cell system, were seeded on the preformed RGD hydrogel constructs and incubated for 48 h at 37 °C and 5% CO_2_.

Imaging of cells on HG-PεK and HG-PEG at a 1:2 molar ratio in the absence of RGD showed high cell viability with a higher number of cells observed in HG-PεK, indicative of the capability of cells to interact with the hydrogel matrix. In contrast, higher numbers of dead cells were observed for HG-PεK with a molar ratio of 1:10, with few cells surviving at higher concentrations of RGD. In terms of cell morphology, ball-like cluster formations with high cell viability were observed in HG-PεK with a molar ratio of 1:2 at all RGD concentrations, but with increasing numbers of dead cells at higher RGD concentrations. Similarly, HG-PEG with a molar ratio of 1:2 gave ball-like cell cluster formations with few dead cells at 0.2 mM RGD, while above this concentration string-like clusters with high cell viability were observed along the strands of the hydrogel network structure, indicative of the efficacy of RGD in promoting cell adhesion (Figure 4 and Figure S4 in the Supplementary Materials).

**Figure 5:**
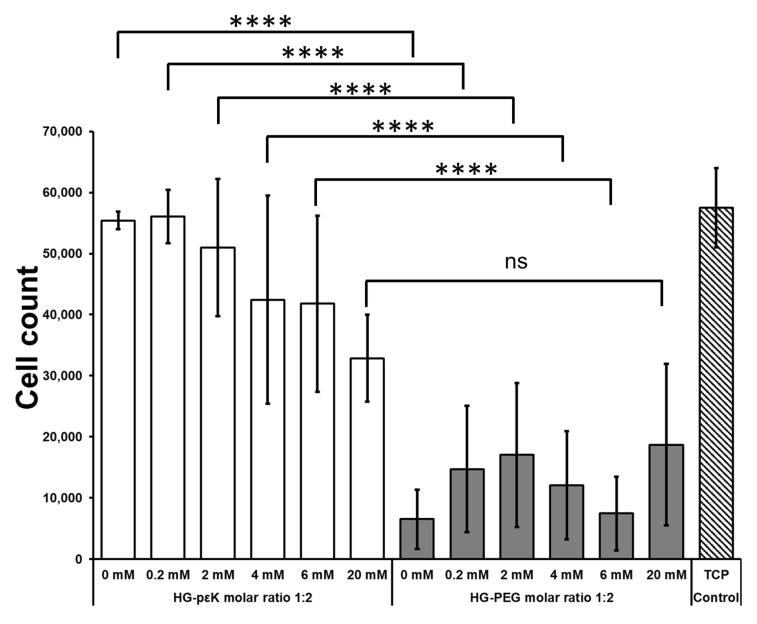
HeLa cell proliferation capability on HG-PεK and HG-PEG with molar ratios of 1:2 and varying levels of RGD. AlamarBlue metabolic activity assay after 48 h cell incubation (see Figures S3–S5 in Supplementary Materials). Tissue culture plastic (TCP) control experiment corresponds to cells seeded on tissue culture plate. Cell count on hydrogels confirmed live/dead cell viability experiments for the HG-PεK and HG-PEG gels. Cell number analysis indicated higher cell proliferation rates on HG-PεK after 48 h compared to cells cultured on the HG-PEG constructs. One-way ANOVA with Bonferroni post-test (ns = no significant and **** *p* ≤ 0.0001). Data represent mean ± standard deviation (*n* = 3).

Additionally, HeLa cell viability/proliferation capability on the hydrogels was assessed using an AlamarBlue assay (Figure 5). 2.0 × 10^4^. HeLa cells per well were seeded on tissue culture plastic (TCP), HG-PεK, and HG-PEG at a molar ratio of 1:2 with differing concentrations of RGD.

On TCP after 48 h incubation time a three-fold cell increase was observed. A similar cell increase was observed for HG-PεK with molar ratio 1:2 in the absence of RGD (no significant difference, *p* > 0.05) while HG-PEG with a molar ratio of 1:2 (without RGD) showed the opposite behavior with a three-fold decrease in cells (significantly different, *p* ≤ 0.0001) compared to TCP and HG-PεK in the absence of RGD. RGD levels in HG-PεK and HG-PEG resulted in significantly different increases in cell number, with HG-PεK having a three-fold increase in cell number (*p* ≤ 0.0001) at 0.2 and 2 mM RGD and a significantly different two-fold cell increase (*p* ≤ 0.0001) at 4 and 6 mM RGD after 48 h. No significant difference was observed between HG-PεK and HG-PEG at 20 mM RGD. These results were indicative of good cell viability and proliferation on HG-PεK within the RGD concentration range analyzed from 0 to 6 mM. On the other hand, no cell proliferation was detected on HG-PEG in line with cell viabilities reported in conventional chemically crosslinked PEG hydrogels containing RGD [58].

Overall, hydrogels containing poly-ε-lysine were successfully formed using dynamic imine crosslinking. High quantities of poly-ε-lysine in the hydrogel formulation improved the storage moduli of gels and gelation time compared to low poly-ε-lysine levels (molar ratio 1:2 relative to crosslinker). In contrast, low levels of poly-ε-lysine improved cell viability but too high a ratio of poly-ε-lysine was detrimental to cell viability. This cytotoxicity at high levels of poly-ε-lysine (molar ratio 1:10 relative to crosslinker) arises due to excess of free amines in the hydrogel scaffold, in agreement with reported cytotoxicity for polymeric biomaterials with high free amine concentrations [50].

Oscillatory rheology and cryo-SEM characterization showed that HG-PεK with a molar ratio of 1:2 formed a stiffer construct compared to HG-PEG: a non-fouling hydrogel broadly used for 3D cell culture. Interestingly, the incorporation of high levels of the *bis*-amine RGD peptide resulted in softer HG-PεK constructs, while the expected crosslinking effect was detected in HG-PEG with increasing levels of RGD leading to stiffer constructs. Despite this unexpected difference in mechanical properties with the incorporation of RGD in the hydrogel formulation, HG-PεK constructs displayed good cytocompatibility with higher metabolic activity/viability detected in HG-PεK with 0 and 0.2 mM of RGD leading to three-fold increase in cell number after 48 h cell culture. Cell adhesion and metabolic activity have been reported in chemically static poly-ε-lysine hydrogels with enhanced stiffness (Young modulus of 0.11 MPa) [51]. Here, these investigations demonstrated that the dynamic crosslinked HG-PεK with molar ratio of 1:2 (storage moduli of 0.02 MPa) formulated with low RGD concentrations provided enough stability and structural support, like chemically static poly-ε-lysine hydrogels, to afford cellular adhesion and proliferation.

## 4. Conclusions

Using reversible Schiff-base bond formation, hydrogels containing poly-ε-lysine and PEG were prepared to evaluate their feasibility for cell culture. The use of poly-ε-lysine proved advantageous because of its simple formulation yielding gels via reversible imine formation using low quantities of material. This is in comparison to other natural polymer precursors that can require further functionalization to produce dynamic crosslinking. HG-PεK with low molar ratios of poly-ε-lysine resulted in suitable candidates for cell culture that displayed good cell adhesion and cytocompatibility. The cell binding RGD peptide was incorporated into the hydrogel network to enhance cell adhesion and biomechanical material properties. The metabolic activity of cells on the biomaterial was measured with the AlamarBlue assay, indicating higher metabolic activity in HG-PεK with low levels of RGD compared to HG-PEG. In this study, dynamic poly-ε-lysine hydrogels were generated with RGD but this approach is highly tunable, for example differing cell adhesive ligands such as laminin could be readily added for cell culture of other mammalian cells.

## Supplementary Materials

The following are available online at https://www.mdpi.com/1996-1944/13/17/3851/s1: Figure S1: ^1^H NMR spectrum 4-arm PEG-aldehydes, Figure S2: FT-IR spectra for 4-arm PEG-OH and 4-arm PEG-aldehydes, Figure S3: Brightfield microscopy of HeLa cell seeded on tissue culture plastic for AlamarBlue assay, Figure S4: Brightfield microscopy of cells seeded at density of 20,000 on dynamic hydrogels and incubated in complete DMEM at 37 °C and 5% CO_2_ for AlamarBlue assay, Figure S5: Calibration curve fluorescence vs. cell number, Figure S6: Images of HG-PεK (molar ratio 1:2) formulated at differing concentrations of 4-arm PEG-aldehydes, Figure S7: Images of HG-PEG (molar ratio 1:2) formulated at differing concentrations of 4-arm PEG-aldehydes, Figure S8: Frequency sweep plots of HG-PεK (molar ratio 1:2) with 0, 2, and 4 mM RGD at constant strain of 1% at 37 °C, Figure S9: Frequency sweep plots of HG-PεK (molar ratio 1:10) with 0, 2, 4, and 6 mM RGD at constant strain of 1% at 37 °C, Figure S10: Frequency sweep plots of HG-PEG (molar ratio 1:2) with 0, 2, 4, and 6 mM RGD at constant strain of 1% at 37 °C, Table S1: Formulation of HG-PεK (molar ratio 1:2) with varying levels of the peptide RGD, Table S2: Formulation of HG-PεK (molar ratio 1:10) with varying levels of the peptide RGD, Table S3: Formulation of HG-PEG (molar ratio 1:2) with varying levels of the peptide RGD.
