# Supplementary material for: Poly-Epsilon-Lysine Hydrogels with Dynamic Crosslinking Facilitates Cell Proliferation
Source: Materials (Basel). 2020 Sep 1;13(17):3851. doi: 10.3390/ma13173851 (PMC7504584; doi:10.3390/ma13173851)
Supplement: Supplementary file 1 [file materials-13-03851-s001.pdf]

materials

Supplementary Materials

Poly-Epsilon-Lysine Hydrogels with Dynamic Crosslinking Facilitates Cell Proliferation

Nestor Lopez Mora, Matthew Owens, Sara Schmidt, Andreia F. Silva and Mark Bradley

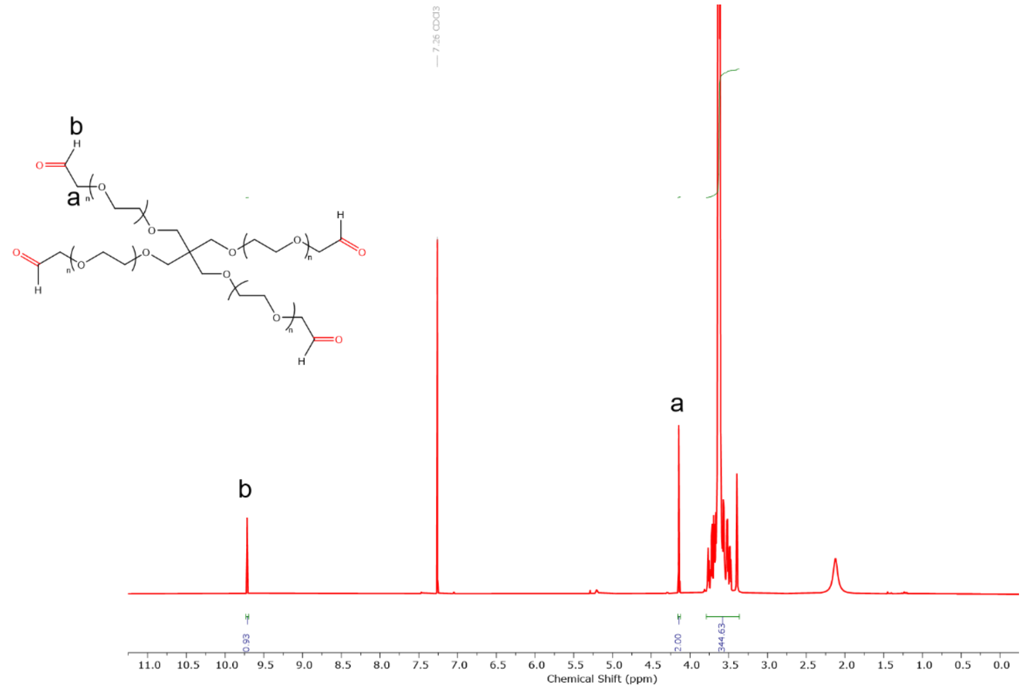

Figure S1. ^{1}H NMR spectrum 4-arm PEG-aldehydes. ($CDCl_3$, 500 MHz). δ 9.7 ppm (s, 1H), δ 4.2 ppm (s, 2H), 3.8–3.5 ppm (m, PEG protons).

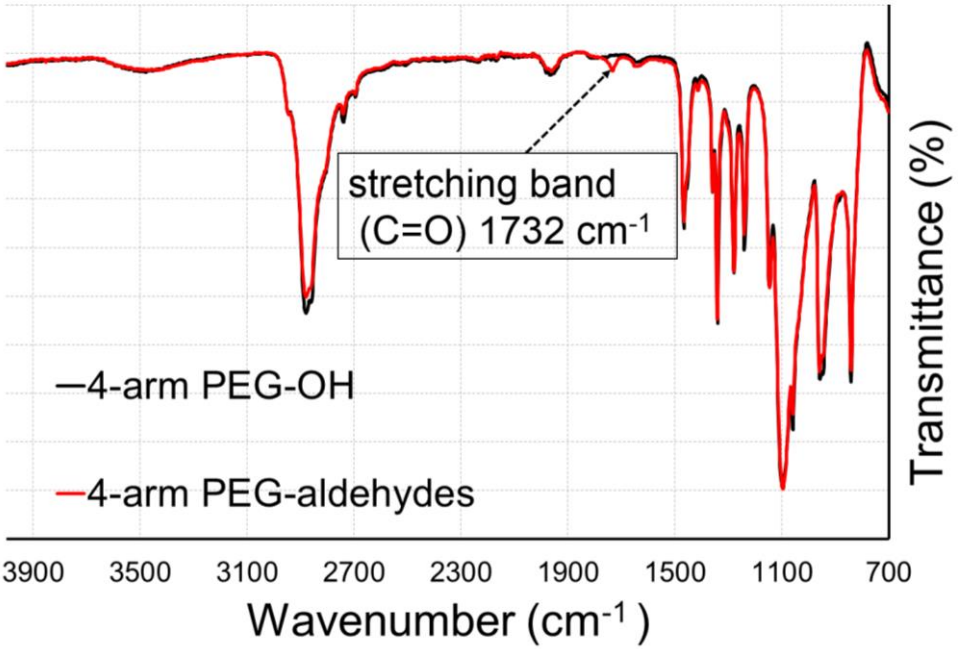

Figure S2. FT-IR spectra for 4-arm PEG-OH and 4-arm PEG-aldehydes. The C=O stretching band at 1732 cm^{-1} indicates the aldehydes functionalization in the 4-arm PEG-OH.

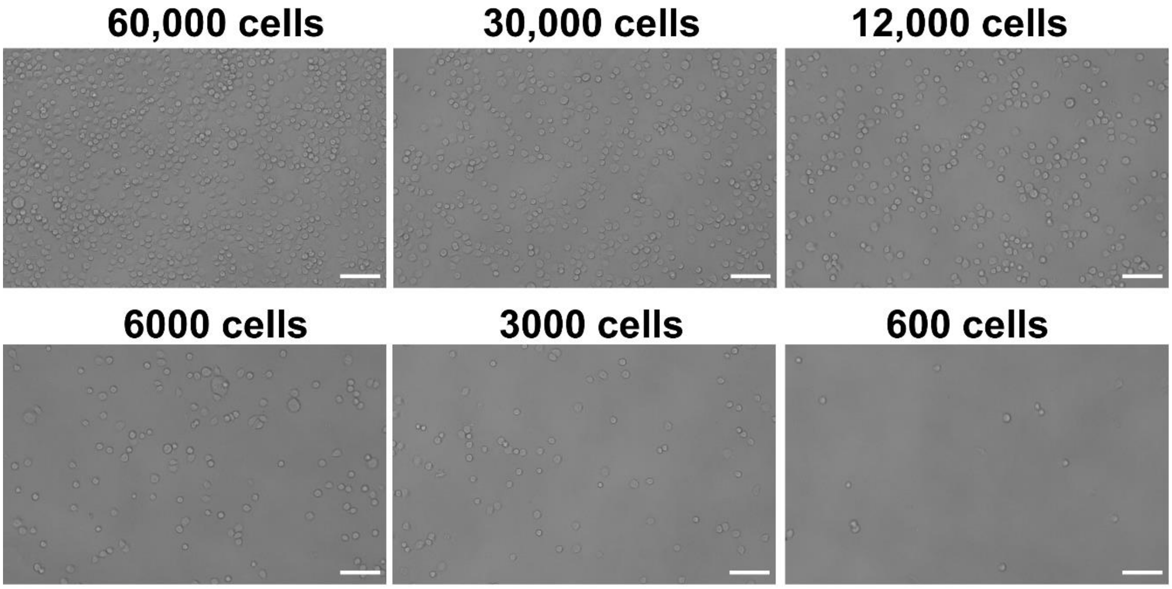

Figure S3. Brightfield microscopy of HeLa cells seeded on the tissue culture plastic at varying cell densities for the construction of the AlamarBlue calibration curve. Scale bars are 200 μm.

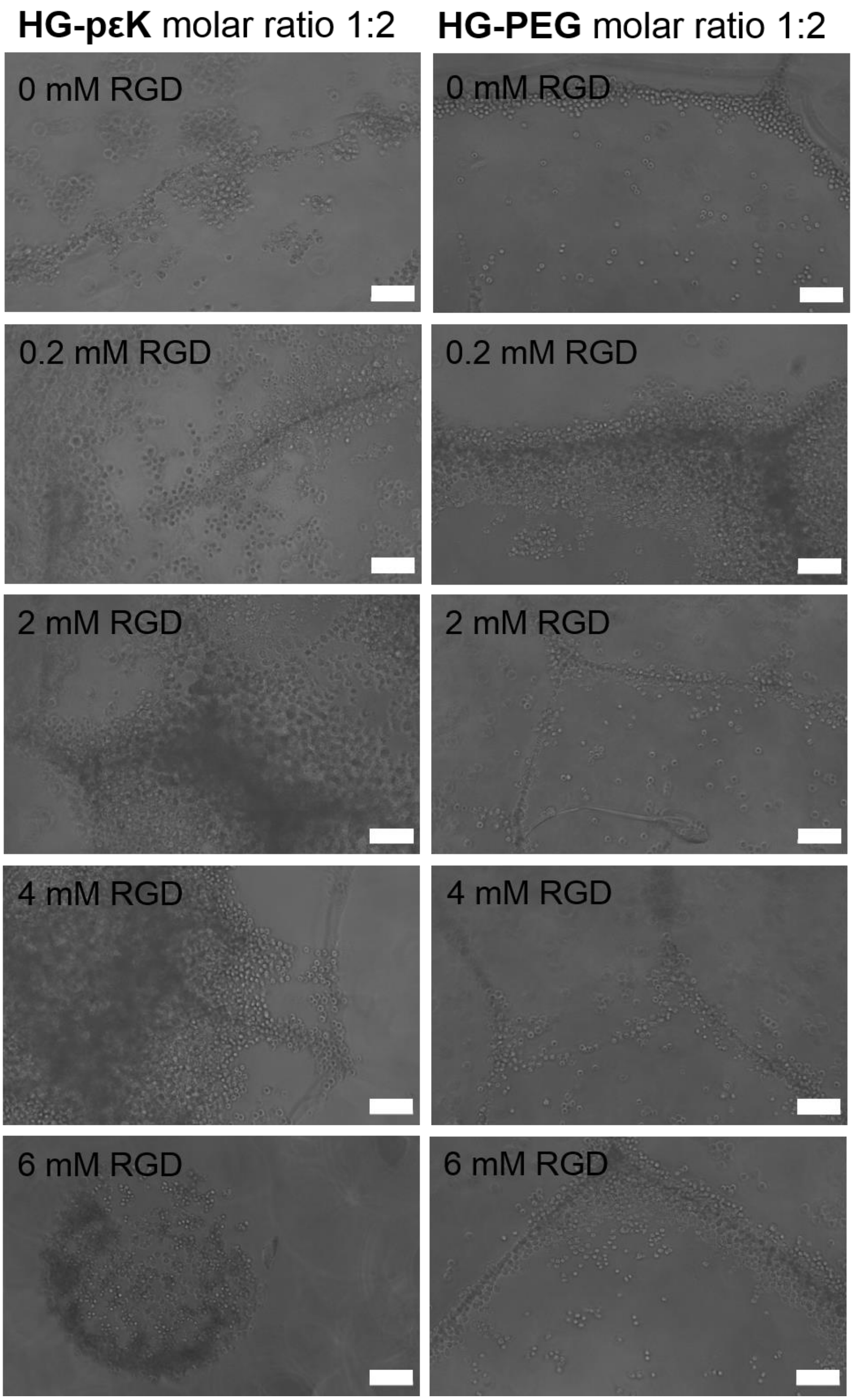

Figure S4. Brightfield microscopy of cells seeded at density of 20,000 on dynamic hydrogels and incubated in complete DMEM at 37 °C and 5% CO_2 AlamarBlue assay. Scale bars are 200 μm.

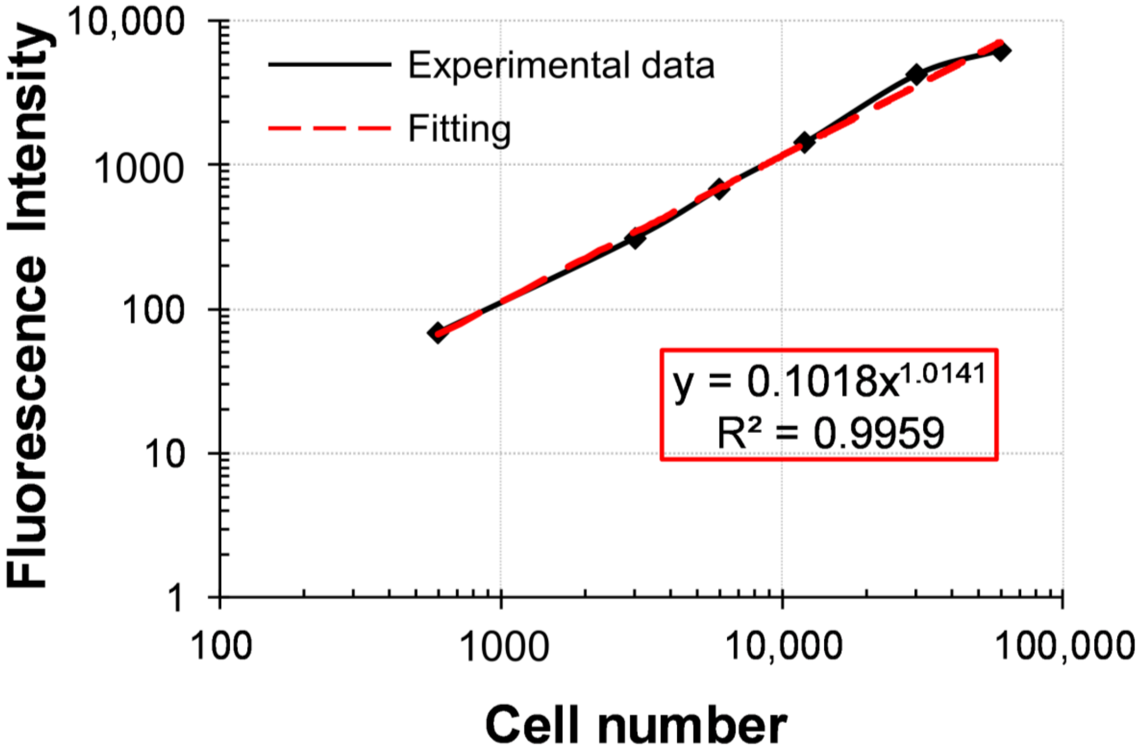

Figure S5. Calibration curve fluorescence *vs* cell number. The fluorescence of AlamarBlue (resazurin) at 590 nm was measured at 5 h. HeLa cell number growing on the library of dynamic hydrogels was quantified using the calibration curve at 5 h (Figure 5 in the main manuscript).

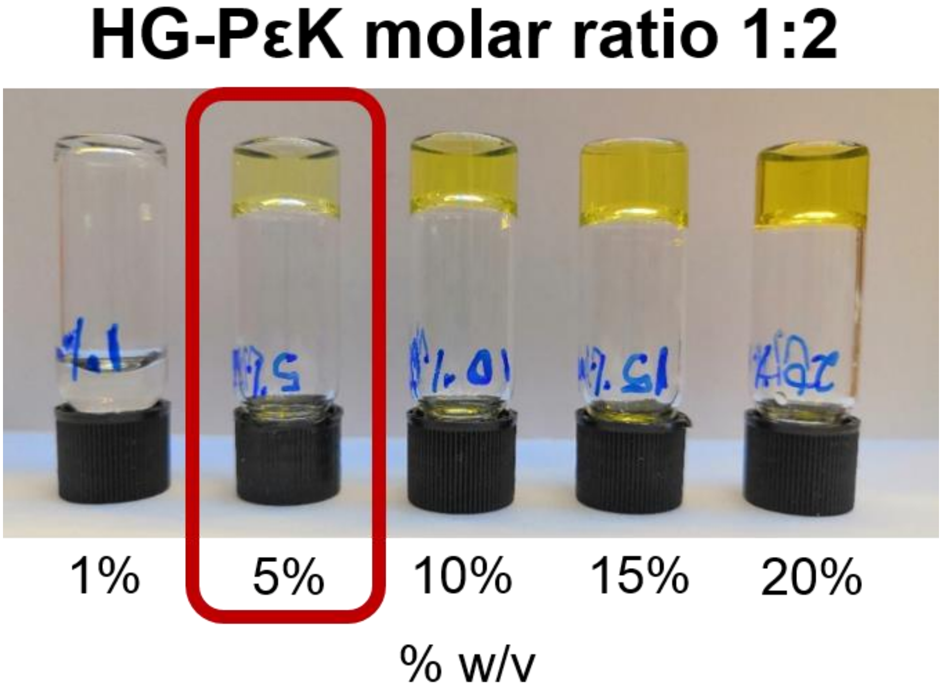

Figure S6. Images of HG-PεK (molar ratio 1:2) formulated using differing concentrations of 4-arm PEG-aldehydes. The concentration of 4-arm PEG-aldehydes necessary for gelation was estimated by inverting the glass vials until the gel solution stopped flowing.

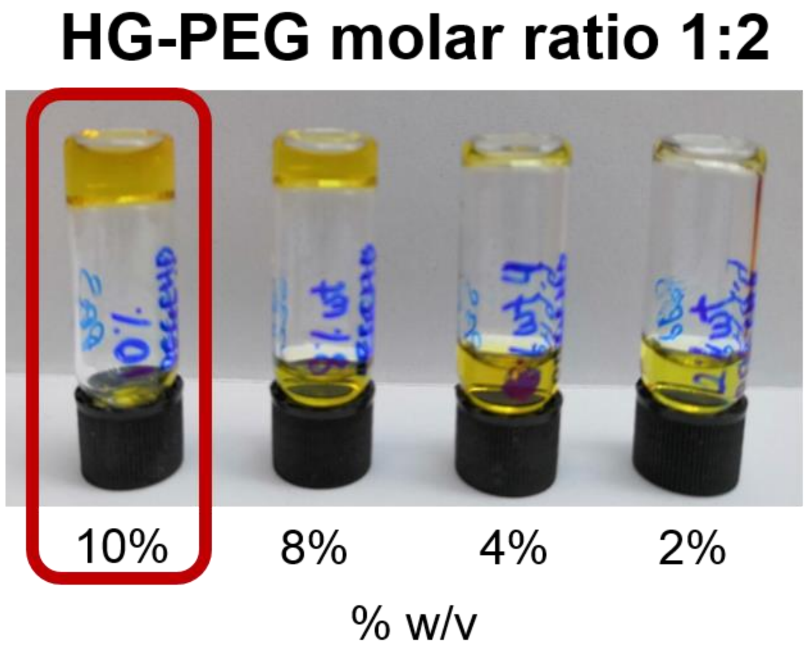

Figure S7. Images of HG-PEG (molar ratio 1:2) formulated at differing concentrations of 4-arm PEG-aldehydes. The concentration of 4-arm PEG-aldehydes necessary for gelation was estimated by inverting the glass vials until the gel solution stopped flowing.

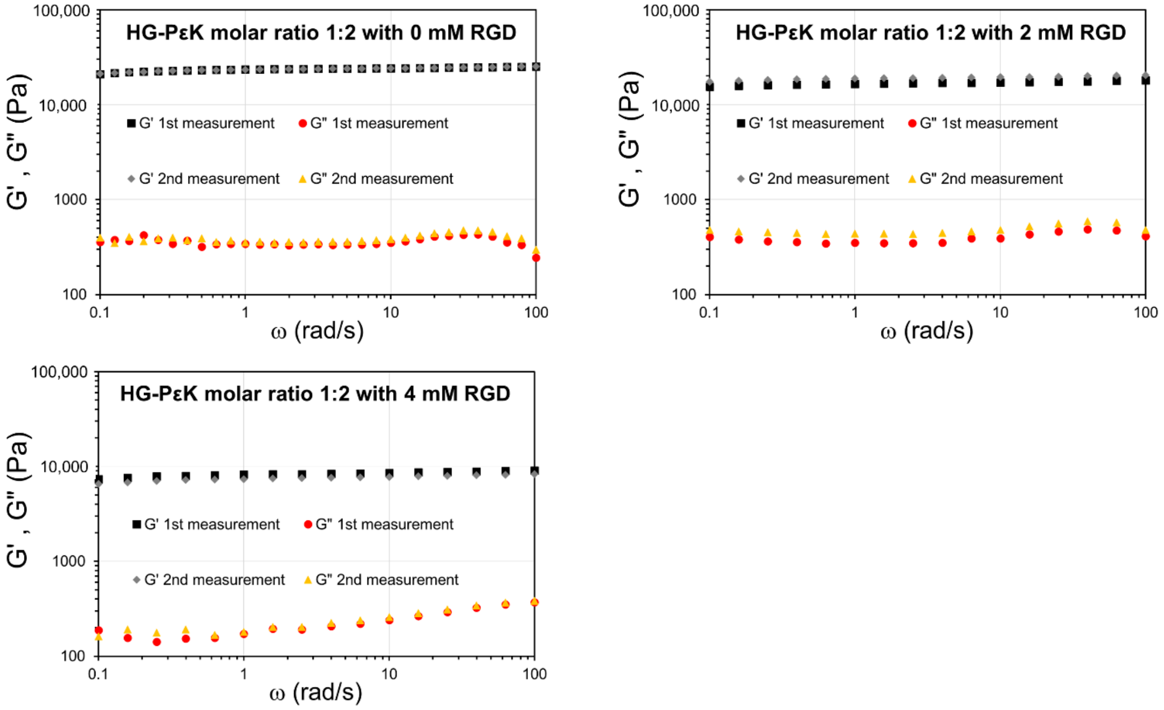

Figure S8. Frequency sweep plots of HG-PεK (molar ratio 1:2) with 0, 2 and 4 mM RGD at constant strain of 1% at 37 °C. The storage moduli (G') at 6.28 rad/s (1 Hz) was used to build Figure 2D in main manuscript. Frequency sweeps were made in duplicate.

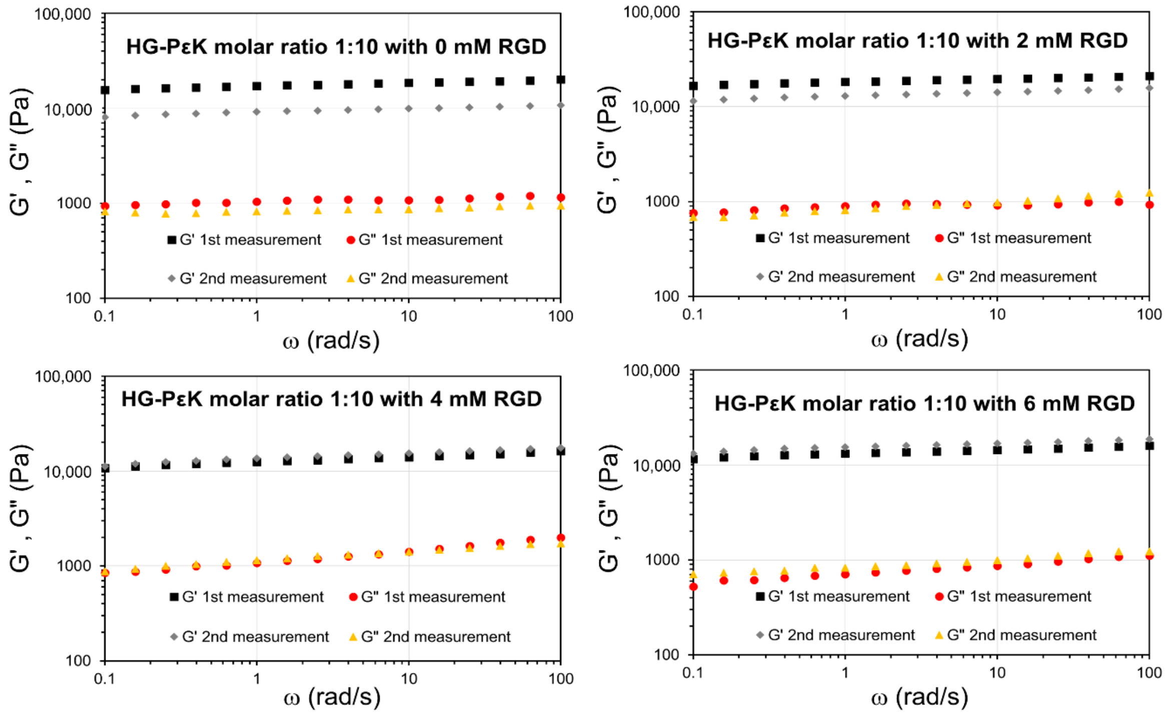

Figure S9. Frequency sweep plots of HG-PεK (molar ratio 1:10) with 0, 2, 4 and 6 mM RGD at constant strain of 1% at 37 °C. The storage moduli (G') at 6.28 rad/s (1 Hz) was used to build Figure 2D in the main manuscript. Frequency sweeps were made in duplicate.

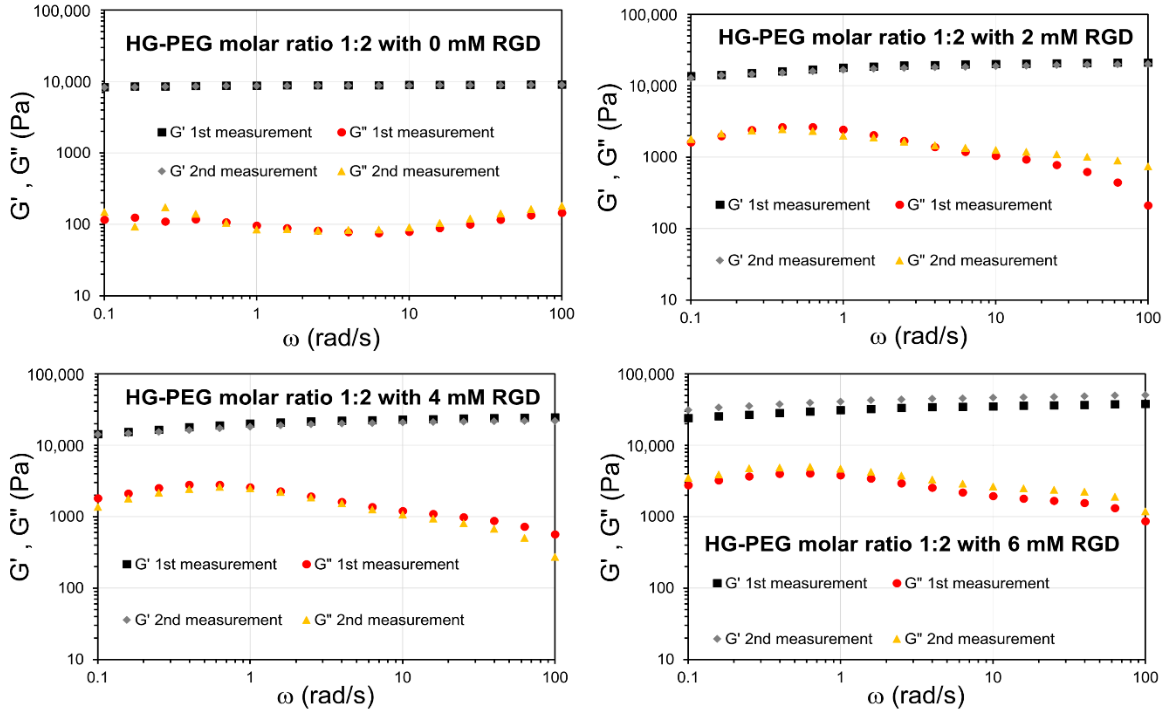

Figure S10. Frequency sweep plots of HG-PEG (molar ratio 1:2) with 0, 2, 4 and 6 mM RGD at constant strain of 1% at 37 °C. The storage moduli (G') at 6.28 rad/s (1 Hz) was used to build Figure 2D in main manuscript. Frequency sweeps were made in duplicate.

Table S1. Formulation of HG-PεK (molar ratio 1:2) with varying levels of the peptide RGD.

**Hydrogel Precursors**	**4-arm PEG-aldehydes**	**Poly-ε-lysine**	**RGD**	**PBS**	**pH**
**Stock Concentration**	**200 mg/mL**	**50 mg/mL**	**100 mM**		
HG-PεK molar ratio 1:2 with 0 mM RGD	250 μL	100 μL	0 μL	150 μL	7
HG-PεK molar ratio 1:2 with 0.2 mM RGD	250 μL	100 μL	1 μL	149 μL	7
HG-PεK molar ratio 1:2 with 2 mM RGD	250 μL	100 μL	10 μL	140 μL	7
HG-PεK molar ratio 1:2 with 4 mM RGD	250 μL	100 μL	20 μL	130 μL	7
HG-PεK molar ratio 1:2 with 6 mM RGD	250 μL	90 μL	30 μL	130 μL	6
HG-PεK molar ratio 1:2 with 20 mM RGD	250 μL	80 μL	100 μL	70 μL	3

Table S2. Formulation of HG-PεK (molar ratio 1:10) with varying levels of the peptide RGD.

**Hydrogel Precursors**	**4-arm PEG-aldehydes**	**poly-ε-lysine**	**RGD**	**PBS**	**pH**
**Stock Concentration**	**200 mg/mL**	**200 mg/mL**	**100 mM**		
HG-PεK molar ratio 1:10 with 0 mM RGD	250 μL	130 μL	0 μL	120 μL	7
HG-PεK molar ratio 1:10 with 0.2 mM RGD	250 μL	130 μL	1 μL	119 μL	7
HG-PεK molar ratio 1:10 with 2 mM RGD	250 μL	130 μL	10 μL	110 μL	7
HG-PεK molar ratio 1:10 with 4 mM RGD	250 μL	130 μL	20 μL	100 μL	7
HG-PεK molar ratio 1:10 with 6 mM RGD	250 μL	130 μL	30 μL	90 μL	6
HG-PεK molar ratio 1:10 with 20 mM RGD	250 μL	120 μL	100 μL	30 μL	3

Note: This pH change for the construct with 20 mM RGD was attributed to the trifluoroacetic acid salt coming from peptide synthesis, that exceeded the PBS buffer capability, thus affecting the Schiff-base bond equilibrium and hydrogel gelation rate at high RGD concentrations.

Table S3. Formulation of HG-PEG (molar ratio 1:2) with varying levels of the peptide RGD.

**Hydrogel Precursors**	**4-arm PEG-aldehydes**	**Diamine PEG**	**RGD**	**PBS**	**pH**
**Stock Concentration**	**200 mg/mL**	**200 mg/mL**	**100 mM**		
HG-PEG molar ratio 1:2 with 0 mM RGD	250 μL	200 μL	0 μL	50 μL	7
HG-PEG molar ratio 1:2 with 0.2 mM RGD	250 μL	200 μL	1 μL	49 μL	7
HG-PEG molar ratio 1:2 with 2 mM RGD	250 μL	195 μL	10 μL	45 μL	7
HG-PEG molar ratio 1:2 with 4 mM RGD	250 μL	190 μL	20 μL	40 μL	6
HG-PEG molar ratio 1:2 with 6 mM RGD	250 μL	185 μL	30 μL	35 μL	6
HG-PEG molar ratio 1:2 with 20 mM RGD	250 μL	150 μL	100 μL	0 μL	3

© 2020 by the authors. Submitted for possible open access publication under the terms and conditions of the Creative Commons Attribution (CC BY) license (http://creativecommons.org/licenses/by/4.0/).
